# Structure-Based Screening Identifies Polyphenol Glucuronides as Cyclophilin D Ligands

**DOI:** 10.3390/molecules31173074

**Published:** 2026-09-01

**Authors:** Annan Wu, Jie Zhang, Jing Zhao

**Affiliations:** 1 College of Food Science and Nutritional Engineering, China Agricultural University, Beijing 100083, China; s20213061005@cau.edu.cn (A.W.); a1515528853@163.com (J.Z.); 2 National Engineering Research Center for Fruit & Vegetable Processing, Beijing 100083, China; 3 Beijing Key Laboratory for Food Non-Thermal Processing, Beijing 100083, China

**Keywords:** Cyclophilin D, mitochondrial permeability transition pore, virtual screening, molecular docking, NMR, surface plasmon resonance, polyphenols

## Abstract

(1) Background: The mitochondrial permeability transition pore (mPTP) contributes to mitochondrial dysfunction under pathological conditions. Cyclophilin D (CypD), a key regulator of sustained mPTP opening, represents an important molecular target. However, systematic strategies for selecting an appropriate CypD crystal structure for virtual screening and efficiently identifying CypD-binding natural polyphenols remain limited. (2) Methods: Six CypD crystal structures were evaluated by receiver operating characteristic analysis using reported CypD inhibitors and approximately 4000 decoys. The optimal receptor structure was subsequently used to screen 484 dietary polyphenols by molecular docking. Selected top-ranked candidates were further characterized by ^15^N–^1^H HSQC NMR titration and surface plasmon resonance (SPR). (3) Results: PDB 6R8L exhibited the best virtual-screening performance, particularly in early enrichment. Several polyphenol glucuronides ranked among the top candidates. NMR analysis showed that apigenin 7-glucuronide and luteolin 7-glucuronide induced pronounced chemical shift perturbations at CypD residues F60, M61, I117, and W121, with F60, M61, and W121 located within or near the docking-predicted S1 binding region. SPR independently confirmed direct binding, yielding *K_D_* values of 66 μM and 84 μM for apigenin 7-glucuronide and luteolin 7-glucuronide, respectively. (4) Conclusions: Integrating systematic receptor evaluation, molecular docking, and complementary NMR and SPR characterization provides a practical workflow for identifying and characterizing natural CypD-binding compounds. Apigenin 7-glucuronide and luteolin 7-glucuronide were identified as micromolar CypD-binding candidates, highlighting polyphenol glucuronides as a chemical class warranting further investigation in CypD ligand discovery.

## 1. Introduction

The mitochondrial permeability transition pore (mPTP) is a non-selective channel located in the inner mitochondrial membrane that plays a critical role in regulating mitochondrial homeostasis. Under pathological conditions, including oxidative stress and calcium overload, prolonged opening of the mPTP results in mitochondrial swelling, membrane depolarization, ATP depletion, and ultimately cell death [1,2,3]. Dysregulated mPTP opening has been implicated in numerous disorders, including ischemia–reperfusion injury, neurodegenerative diseases, metabolic disorders, and liver diseases [4,5,6,7]. Therefore, preventing sustained mPTP opening has become an attractive therapeutic strategy.

Among the regulatory components of the mPTP, Cyclophilin D (CypD) is regarded as the most extensively validated pharmacological target because it regulates pore opening through its peptidyl-prolyl cis-trans isomerase activity [1,8]. Structurally, CypD contains a conserved catalytic pocket and a substrate-binding groove, which provide a molecular basis for structure-guided inhibitor discovery [9]. Genetic deletion or pharmacological inhibition of CypD markedly suppresses mPTP opening and alleviates mitochondrial dysfunction in multiple disease models. Consequently, considerable efforts have been devoted to developing selective CypD inhibitors.

Cyclosporin A and several synthetic derivatives remain among the most representative CypD inhibitors [8,10,11]. However, their therapeutic application is limited by insufficient selectivity, poor bioavailability, and immunosuppressive effects [8,12]. Natural products, particularly dietary polyphenols and their metabolites, offer substantial structural diversity, generally favorable safety profiles, and broad biological activities, making them attractive sources for CypD ligand discovery [13,14,15,16,17,18]. Structure-based virtual screening provides an efficient means of exploring such chemically diverse compound libraries; however, its predictive performance is highly dependent on the receptor conformation used for docking. Conventional virtual screening often relies on a single protein structure, whereas differences in binding-pocket geometry and side-chain orientation among available crystal structures can substantially affect ligand ranking and screening accuracy [19]. Although multiple CypD crystal structures are currently available, their relative suitability for virtual screening has not been systematically evaluated. The absence of a rational receptor-selection strategy may therefore limit the reliability and efficiency of structure-based screening for CypD-binding compounds.

To address this methodological gap, we established an integrated workflow combining systematic receptor evaluation, structure-based virtual screening, and complementary biophysical characterization for the identification of natural CypD-binding polyphenols. Candidate CypD crystal structures were first benchmarked by receiver operating characteristic (ROC) analysis using known active compounds and decoys to identify a suitable receptor structure for virtual screening. Dietary polyphenols from the Phenol-Explorer database were subsequently screened by molecular docking, and selected top-ranked candidates were experimentally characterized using ^15^N–^1^H HSQC NMR and surface plasmon resonance (SPR). NMR was used to provide residue-level information on ligand-induced perturbations, whereas SPR independently confirmed direct binding and enabled quantitative determination of binding affinity. Together, this workflow provides a rational framework for receptor selection and integrates computational screening with complementary experimental approaches for the identification and characterization of CypD-binding natural polyphenols.

## 2. Results

### 2.1. Selection of the Optimal CypD Structure for Virtual Screening

The accuracy of structure-based virtual screening largely depends on the selection of an appropriate receptor conformation [20,21]. To identify the most suitable CypD structure for molecular docking, six experimentally determined crystal structures (PDB IDs: 6R8L, 4J59, 5CCS, 2Z6W, 7R2L, and 3R4G) were evaluated using receiver operating characteristic (ROC) analysis. A benchmark dataset consisting of 80 reported CypD inhibitors and approximately 4000 decoy compounds was docked against each structure, and the screening performance was assessed using the area under the ROC curve (AUC), hit rate within the top 0.5% of ranked compounds (HR_0.5%_), and enrichment factor within the top 0.5% (EF_0.5%_).

**Figure 1:**
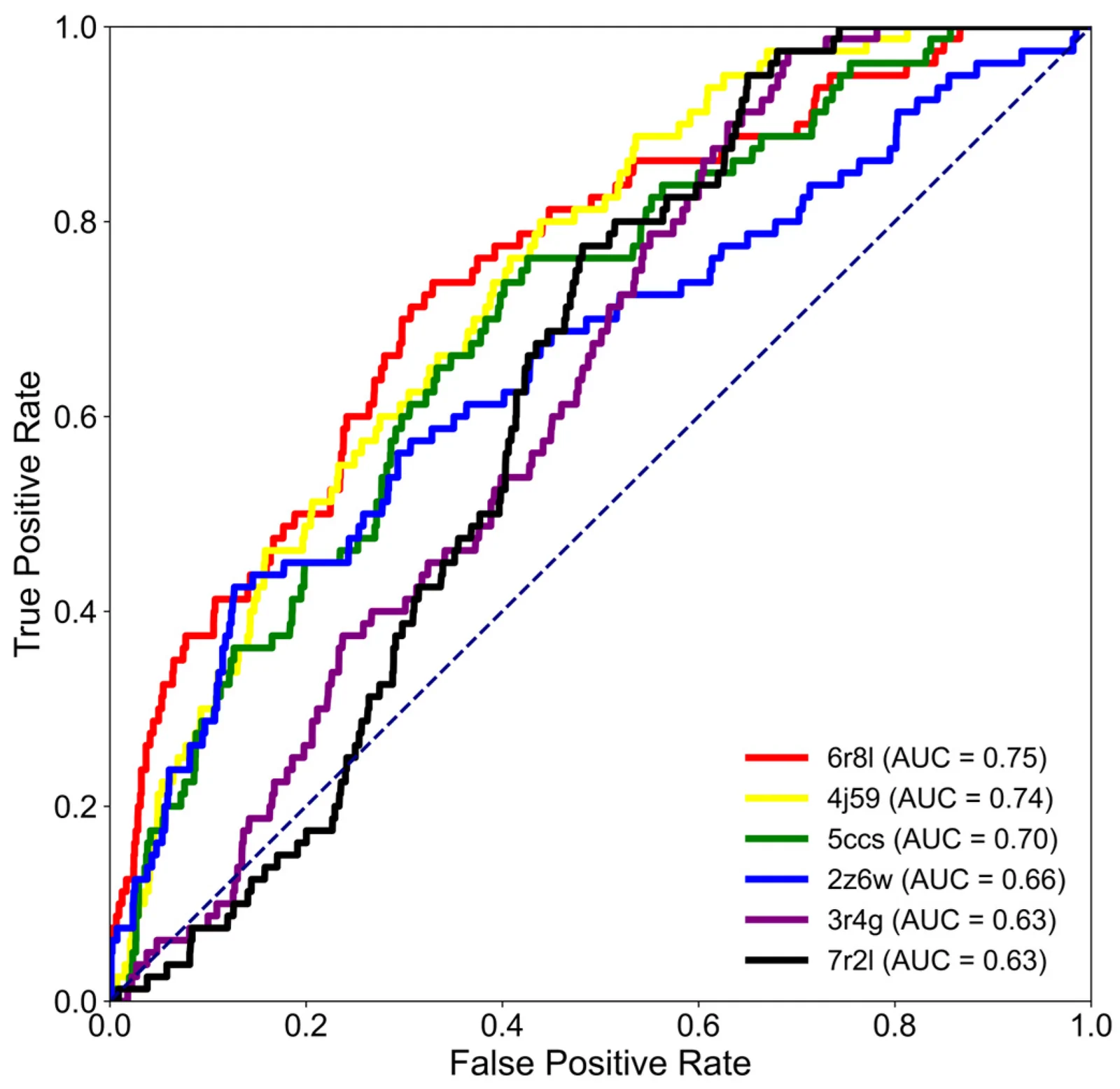
ROC curves for the 6 CypD structures. The AUC values are 0.75 (6R8L), 0.74 (4J59), 0.70 (5CCS), 0.66 (2Z6W), 0.63 (7R2L), and 0.63 (3R4G), respectively. The dashed line represents random classification (AUC = 0.5).

As shown in Figure 1, all six crystal structures exhibited acceptable discrimination between active compounds and decoys, although their screening performances differed considerably. Among them, the 6R8L structure achieved the highest AUC value (0.75), followed by 4J59 (0.74), 5CCS (0.70), 2Z6W (0.66), 7R2L (0.63), and 3R4G (0.63). These results indicate that the different crystallographic conformations of CypD substantially influence virtual screening performance. Because practical virtual screening prioritizes the identification of active compounds within the highest-ranked candidates rather than overall classification performance, early enrichment metrics were further evaluated. As summarized in Table 1, 6R8L exhibited the highest HR_0.5%_ (0.088) and EF_0.5%_ (17.8), whereas the remaining crystal structures showed substantially lower enrichment efficiencies. Although the AUC values of 6R8L and 4J59 were comparable, 4J59 exhibited markedly inferior early enrichment (HR_0.5%_ = 0.013; EF_0.5%_ = 2.5). Likewise, the remaining four structures failed to enrich active compounds efficiently within the top-ranked molecules despite exhibiting moderate AUC values. These findings demonstrate that AUC alone is insufficient for selecting the optimal receptor for virtual screening and highlight the importance of incorporating early-recognition metrics.

Considering the overall discrimination ability together with the early enrichment performance, the 6R8L crystal structure was selected for subsequent molecular docking of dietary polyphenols.

### 2.2. Virtual Screening Identifies Polyphenol Glucuronides as Candidate CypD Ligands

Following selection of the optimal receptor structure (6R8L), a library of 484 dietary polyphenols from the Phenol-Explorer database was subjected to structure-based virtual screening. Prior to large-scale docking, the reliability of the docking protocol was evaluated by re-docking the co-crystallized ligand into the CypD binding pocket. The root-mean-square deviation (RMSD) between the docked and crystal conformations was 0.287 Å, which was well below the generally accepted threshold of 2.0 Å, confirming the reliability of the docking procedure for subsequent virtual screening. The docking results are summarized in Table 2. Apigenin 7-glucuronide exhibited the highest predicted binding affinity (−10.41 kcal/mol), exceeding that of the positive control (the 6R8L-bound ligand; −10.16 kcal/mol). Other highly ranked compounds included 6″-O-acetylglycitin (−9.90 kcal/mol), luteolin 7-glucuronide (−9.70 kcal/mol), ellagic acid glucoside (−9.60 kcal/mol), and 5,3′,4′-trihydroxy-3-methoxy-6,7-methylenedioxyflavone 4′-O-glucuronide (−9.59 kcal/mol). Notably, the highest-ranked candidates were predominantly glycosylated polyphenols, including three glucuronide conjugates, suggesting that glucuronide moieties may contribute to interactions with the CypD binding pocket.

**Figure 2:**
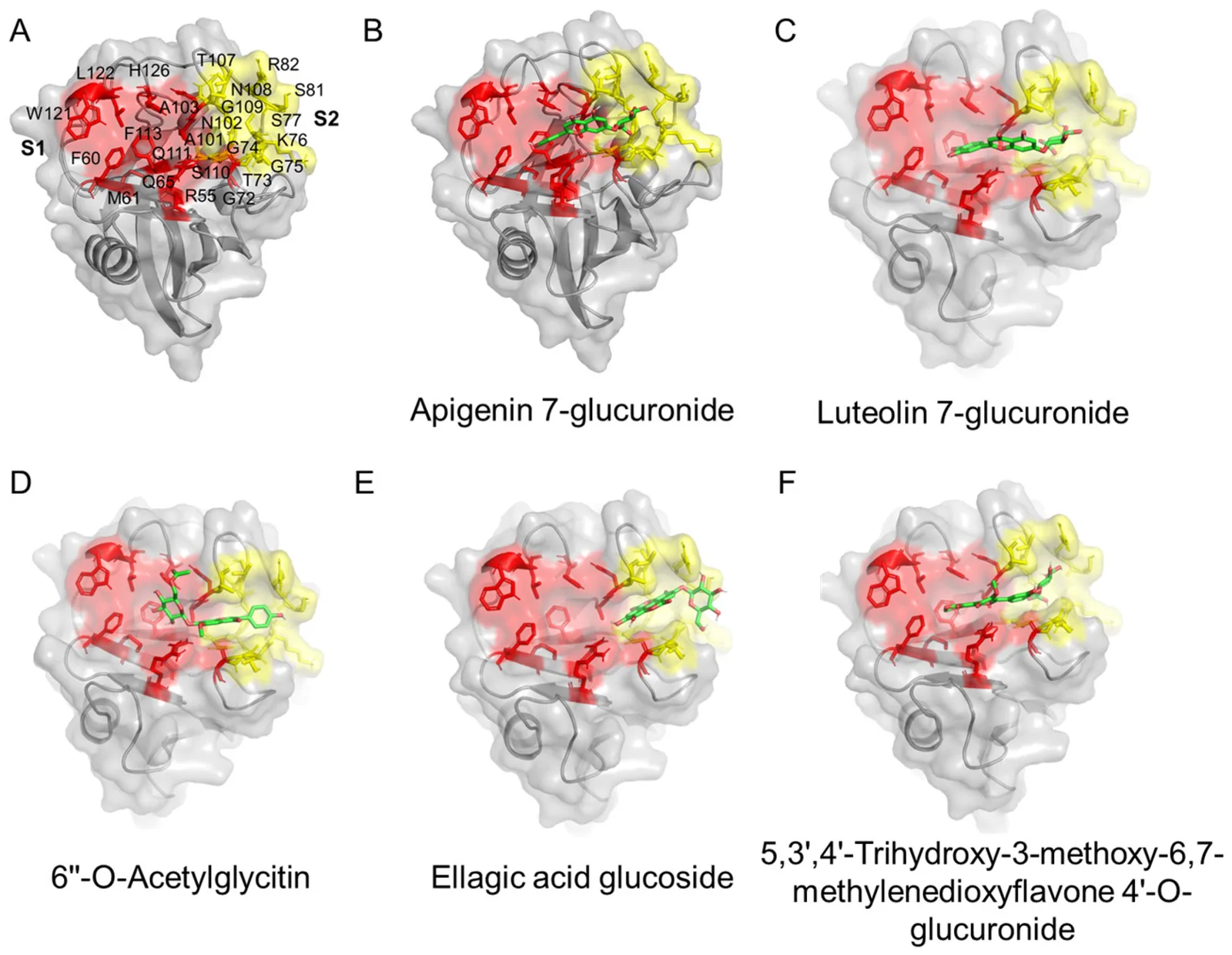
Binding pocket of CypD and binding pose of polyphenols with CypD. (**A**) The active site (S1, red) of CypD is characterized by residues R55, F60, M61, Q63, G72, A101, N102, F113, W121, L122, and H126. Furthermore, it features a substrate-binding pocket (S2, yellow), composed of residues T73, G74, G75, K76, S77, S81, R82, A103, T107, N108, G109, S110, and Q111, which contributes to the formation of the substrate-binding groove. (**B**) Binding Pose of CypD and Apigenin 7-glucuronide. (**C**) Binding Pose of CypD and Luteolin 7-glucuronide. (**D**) Binding Pose of CypD and 6″-O-Acetylglycitin. (**E**) Binding Pose of CypD and Ellagic acid glucoside. (**F**) Binding Pose of CypD and 5,3′,4′-Trihydroxy-3-methoxy-6,7-methylenedioxyflavone 4′-O-glucuronide.

**Figure 3:**
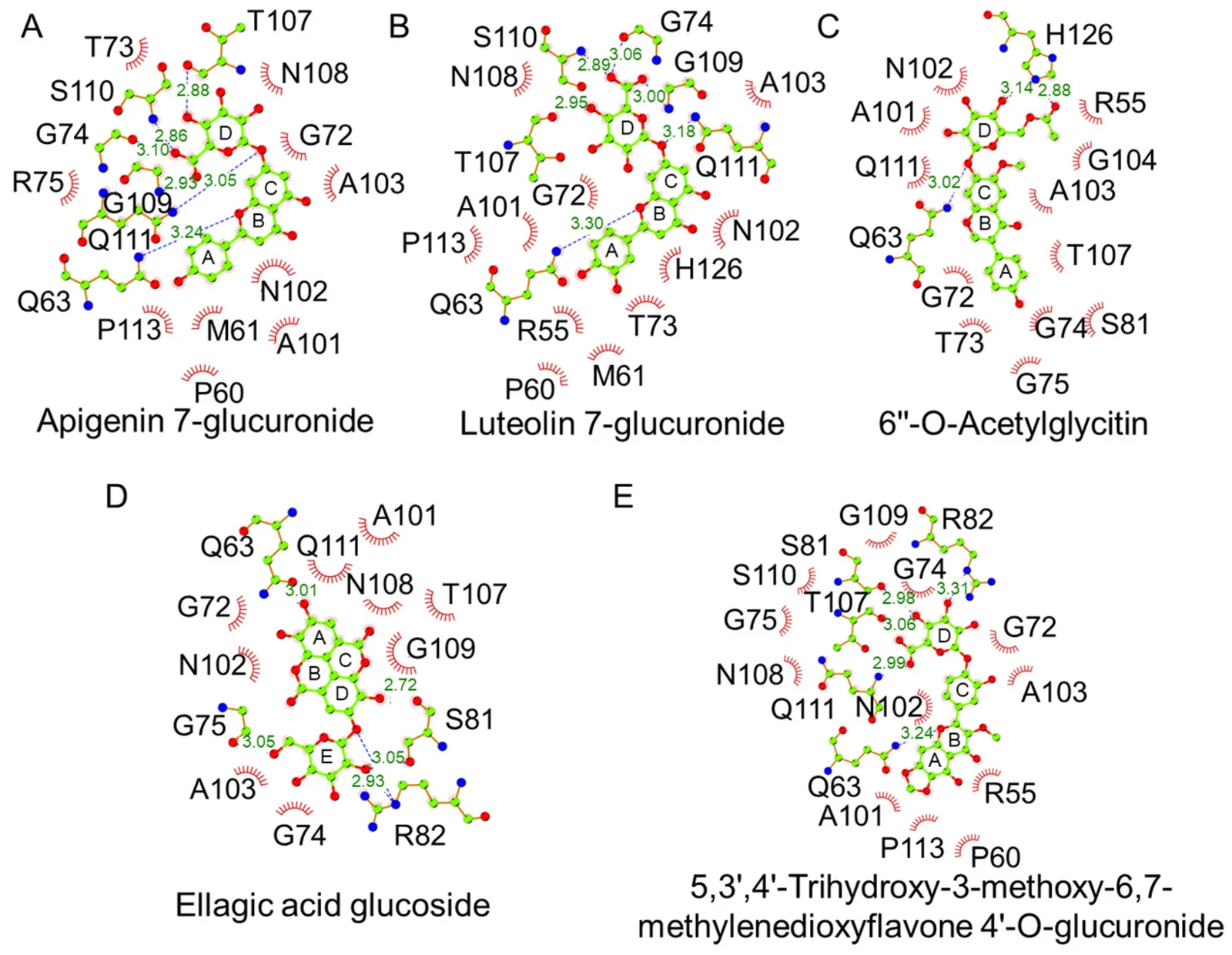
Predicted interactions between the selected polyphenols and CypD. (**A**) Apigenin 7-glucuronide’s B-ring forms hydrogen bonds with Gln63, and the D-ring (glycosidic ring) forms hydrogen bonds with G74, T107, G109, S110, and Q111. Additionally, it interacts hydrophobically with R55, F60, M61, G72, T73, A101, N102, A103, N108, and F113. (**B**) Similar with Apigenin 7-glucuronide, Luteolin 7-glucuronide’s B-ring forms hydrogen bonds with Q63, and the D-ring (glycosidic ring) forms hydrogen bonds with G74, T107, G109, S110, and Q111. Additionally, it interacts hydrophobically with R55, F60, M61, G72, T73, A101, N102, A103, N108, F113 and H126. (**C**) The D-ring (glycosidic ring) of 6″-O-Acetylglycitin forms hydrogen bonds with T73 and H126, and it also interacts hydrophobically with R55, G72, T73, G74, G75, S81, A101, N102, A103, G104, T107, and Q111. (**D**) Ellagic acid glucoside’s A-ring forms hydrogen bonds with Q63, the D-ring forms hydrogen bonds with S81, and the E-ring (glycosidic ring) forms hydrogen bonds with G75 and R82. Additionally, it interacts hydrophobically with G72, G74, A101, N102, A103, T107, N108, G109, and Q111. (**E**) The B-ring of 5,3′,4′-Trihydroxy-3-methoxy-6,7-methylenedioxyflavone 4′-O-glucuronide forms hydrogen bonds with Q63, and the D-ring forms hydrogen bonds with S81, R82, T107, and Q111. Moreover, it interacts hydrophobically with R55, F60, G72, G74, G75, A101, N102, A103, N108, G109, S110, and F113.

To further investigate their binding modes, the docking poses of the top-ranked compounds were analyzed (Figure 2). Apigenin 7-glucuronide, luteolin 7-glucuronide, 6″-O-acetylglycitin, and 5,3′,4′-trihydroxy-3-methoxy-6,7-methylenedioxyflavone 4′-O-glucuronide simultaneously occupied both the catalytic S1 pocket and the adjacent S2 substrate-binding region. In contrast, ellagic acid glucoside primarily interacted with residues within the S2 region. These results indicate that simultaneous engagement of both binding regions may contribute to the stronger predicted binding affinities observed for the top-ranked compounds. Protein–ligand interaction analysis further revealed a common interaction pattern among the highest-ranked polyphenol glucuronides (Figure 3). The polyphenol scaffold predominantly established hydrophobic contacts with residues in the catalytic S1 pocket, whereas the glucuronide moiety formed multiple hydrogen bonds with residues located in the S2 region. Apigenin 7-glucuronide and luteolin 7-glucuronide displayed highly similar interaction profiles, involving hydrophobic interactions with residues including R55, F60, M61, G72, A101, N102, A103, and F113, together with hydrogen bonding to residues within the substrate-binding region. Because these two compounds exhibited both high docking scores and highly similar binding modes, they were selected for subsequent experimental validation by ^15^N–^1^H HSQC NMR.

### 2.3. HSQC NMR and SPR Characterization of Polyphenol Glucuronide Binding to CypD

**Figure 4:**
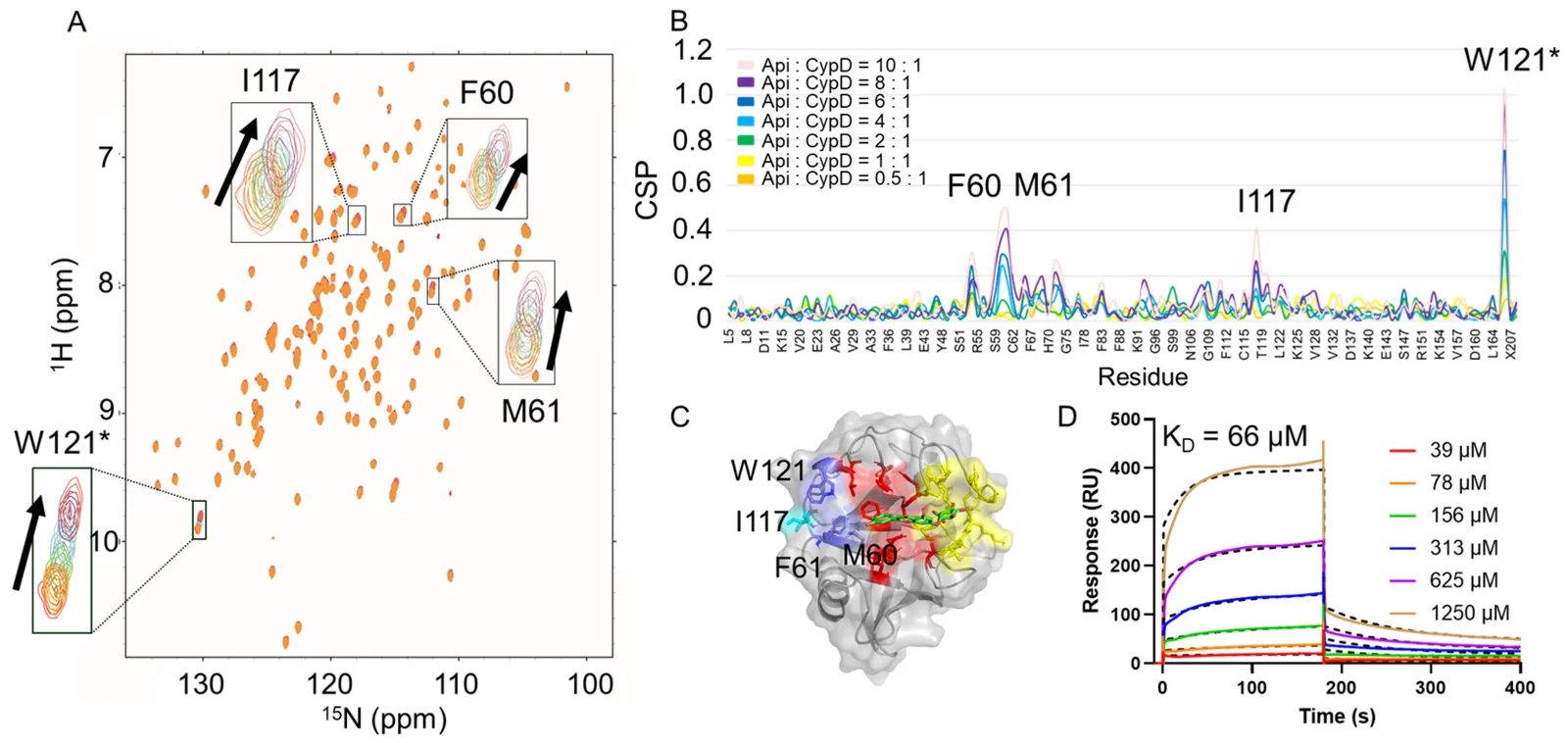
Apigenin 7-glucuronide interacts with CypD at residues F60, M61, I117, and W121. (**A**) Overlay of ^15^N–^1^H HSQC spectra of 0.1 mM CypD titrated with apigenin 7-glucuronide at different molar ratios. The spectra are color-coded according to the apigenin 7-glucuronide: CypD molar ratio: 10:1 (light pink), 8:1 (purple), 6:1 (blue), 4:1 (cyan), 2:1 (green), 1:1 (yellow), and 0.5:1 (orange). Arrows indicate the direction of peak changes upon titration with apigenin 7-glucuronide. (**B**) Residue-specific chemical shift perturbations (CSPs) calculated according to Equation (3), using the same color coding as in (**A**), showing pronounced perturbations at F60, M61, I117, and W121. (**C**) Mapping of the perturbed residues onto the CypD structure shows that F60, M61, and W121 (purple) are located within the S1 pocket, whereas I117 (blue) lies outside the active site. The S1 and S2 pockets are highlighted in red and yellow, respectively. (**D**) SPR sensorgrams for the binding of apigenin 7-glucuronide to CypD. Colored solid lines represent the experimental responses at analyte concentrations of 39, 78, 156, 313, 625, and 1250 μM, while black dashed lines represent the global fits using a two-state binding model. The fitted *K_D_* value was 66 μM. * This is the N-H peak of the side-chain pyrrole ring in W121.

**Figure 5:**
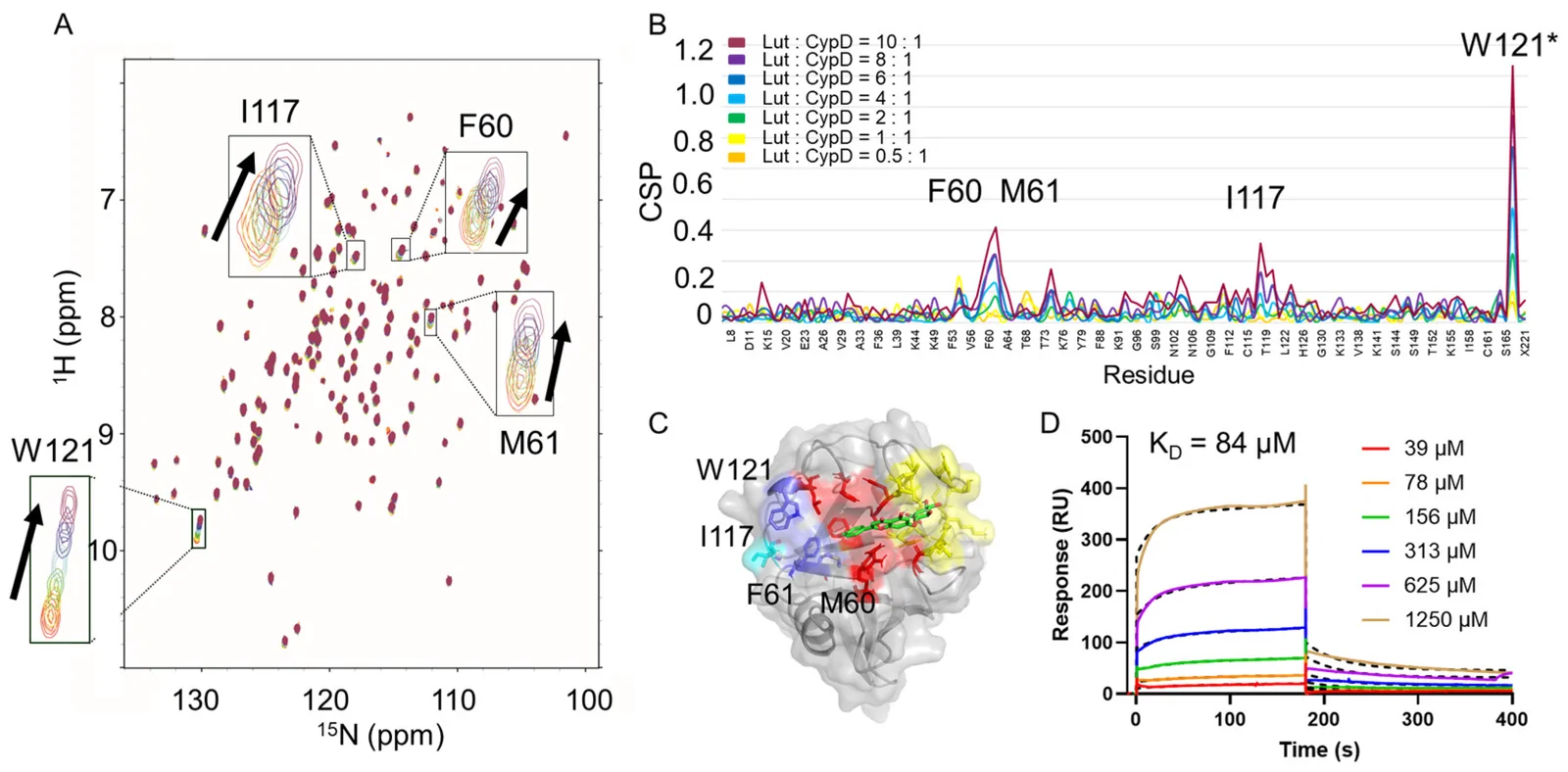
Luteolin 7-glucuronide interacts with CypD at residues F60, M61, I117, and W121. (**A**) Overlay of ^15^N–^1^H HSQC spectra of 0.1 mM CypD titrated with luteolin 7-glucuronide at different molar ratios. The spectra are color-coded according to the luteolin 7-glucuronide:CypD molar ratio: 10:1 (dark red), 8:1 (purple), 6:1 (blue), 4:1 (cyan), 2:1 (green), 1:1 (yellow), and 0.5:1 (orange). Arrows indicate the direction of peak changes upon titration with luteolin 7-glucuronide. (**B**) Residue-specific chemical shift perturbations (CSPs) calculated according to Equation (3), using the same color coding as in (**A**), showing pronounced perturbations at F60, M61, I117, and W121. (**C**) Mapping of the perturbed residues onto the CypD structure shows that F60, M61, and W121 (purple) are located within the S1 pocket, whereas I117 (blue) lies outside the active site. The S1 and S2 pockets are highlighted in red and yellow, respectively. (**D**) SPR sensorgrams for the binding of luteolin 7-glucuronide to CypD. Colored solid lines represent the experimental responses at analyte concentrations of 39, 78, 156, 313, 625, and 1250 μM, while black dashed lines represent the global fits using a two-state binding model. The fitted *K_D_* value was 84 μM. * This is the N-H peak of the side-chain pyrrole ring in W121.

^15^N–^1^H HSQC NMR titration was performed to characterize the interactions of apigenin 7-glucuronide and luteolin 7-glucuronide with CypD at the residue level. Each compound was titrated into CypD at ligand-to-protein molar ratios of 0:1, 0.5:1, 1:1, 2:1, 4:1, 6:1, 8:1, and 10:1. Both compounds induced pronounced chemical shift perturbations (CSPs) at F60, M61, I117, and W121 (Figure 4B and Figure 5B), indicating similar patterns of CypD residues affected upon ligand binding.

To independently confirm the interactions and quantitatively determine their binding affinities, SPR measurements were subsequently performed. Concentration-dependent SPR responses were observed for both compounds, and global fitting using a two-state binding model yielded *K_D_* values of 66 μM for apigenin 7-glucuronide and 84 μM for luteolin 7-glucuronide (Figure 4D and Figure 5D, respectively). These results independently confirm direct binding of both polyphenol glucuronides to CypD and place their affinities in the micromolar range. The similar SPR-derived *K_D_* values further indicate that the two structurally related compounds exhibit comparable affinities for CypD under the experimental conditions used.

Taken together, the NMR and SPR measurements provide complementary information on the CypD–ligand interactions. NMR identifies the CypD residues whose local chemical environments are perturbed upon ligand binding, whereas SPR provides an independent quantitative assessment of binding affinity. The correspondence between the NMR-perturbed residues and the docking-predicted S1 binding region, together with the SPR-derived micromolar affinities, supports direct interactions of apigenin 7-glucuronide and luteolin 7-glucuronide with CypD.

## 3. Discussion

CypD is an important regulator of mitochondrial permeability transition and has emerged as a potential therapeutic target in mitochondria-related disorders [1,8]. Because the performance of structure-based virtual screening is strongly influenced by receptor conformation [20,21,22], six CypD crystal structures were benchmarked using known active compounds and decoys. Although several structures exhibited comparable overall discrimination as assessed by AUC, 6R8L showed the best early enrichment performance. This finding highlights the importance of considering not only global discrimination but also the ability to prioritize active compounds among the highest-ranked candidates when selecting receptor structures for virtual screening. Given the therapeutic relevance of CypD and the limitations associated with currently available inhibitors [17], screening structurally diverse natural compounds may provide an additional source of candidate CypD-binding molecules.

Screening of 484 dietary polyphenols identified several glycosylated compounds among the top-ranked candidates. Docking analysis suggested a common interaction pattern in which the polyphenol scaffold predominantly occupied the hydrophobic S1 pocket, while the glycosidic moieties extended toward the adjacent S2 region and formed additional predicted polar interactions. Structurally, the hydroxyl-rich sugar or glucuronide groups provide multiple potential hydrogen-bonding sites [23], whereas the aromatic polyphenol scaffold can support hydrophobic and van der Waals contacts. Such a combination of polar and nonpolar interaction potentials may be compatible with the relatively large and shallow binding region of CypD [9] and may contribute to molecular recognition of these conjugated polyphenols. Nevertheless, these interactions are derived from docking models and should be regarded as structural hypotheses rather than direct evidence of specific intermolecular contacts or binding affinity.

Experimental characterization using ^15^N–^1^H HSQC NMR provided residue-level evidence for the interactions of apigenin 7-glucuronide and luteolin 7-glucuronide with CypD. Both compounds induced pronounced chemical shift perturbations at F60, M61, I117, and W121. F60, M61, and W121 are located within or near the S1 region, providing experimental support for the involvement of the docking-predicted binding region. In contrast, the perturbation observed for I117 may arise either from proximity to the ligand or from an indirect conformational or environmental change upon binding. Thus, the spatial correspondence between the docking-predicted binding region and the NMR-perturbed residues provides complementary support for the proposed CypD–ligand interactions, but does not establish a unique or definitive binding pose. Because saturation was not achieved within the concentration range accessible in the NMR titrations, the resulting NMR-based affinity estimates were subject to substantial uncertainty [24]. We therefore further characterized the interactions using SPR, which independently confirmed direct binding and yielded *K_D_* values of 66 μM for apigenin 7-glucuronide and 84 μM for luteolin 7-glucuronide. These results place the interactions in the micromolar rather than millimolar range and provide a more reliable quantitative assessment of binding affinity under the present experimental conditions. The similar SPR-derived affinities of the two compounds also suggest that the additional 3′-hydroxyl group of luteolin 7-glucuronide does not markedly alter its overall affinity for CypD. Compared with established CypD ligands and inhibitors, which have generally undergone extensive biochemical and functional characterization [9,10], apigenin 7-glucuronide and luteolin 7-glucuronide should currently be regarded as moderate-affinity CypD-binding candidates rather than validated CypD inhibitors. Their micromolar affinities indicate measurable direct interactions with CypD but do not, by themselves, establish functional potency. Nevertheless, their identification from a dietary polyphenol library expands the structural diversity of compounds reported to interact with CypD and suggests that glucuronidated polyphenol scaffolds may represent a previously underexplored chemical space for CypD ligand discovery. Further comparison with established CypD inhibitors through enzymatic and cellular assays will be necessary to determine whether these interactions translate into functional inhibition.

Several limitations should nevertheless be considered when interpreting these findings. Although NMR and SPR provide complementary evidence that apigenin 7-glucuronide and luteolin 7-glucuronide directly interact with CypD, binding alone does not establish inhibition of CypD activity or regulation of mitochondrial permeability transition. Moreover, the specific hydrogen-bonding interactions predicted for the glucuronide moieties have not been directly resolved experimentally, and the contributions of individual functional groups to CypD recognition remain unclear. Mutational analysis, structure–activity relationship studies, and higher-resolution structural approaches will be required to define these interactions more precisely. In addition, only two top-ranked candidates were experimentally characterized, and validation of a broader set of compounds will be necessary to further assess the predictive performance and generalizability of the screening workflow.

## 4. Materials and Methods

### 4.1. Materials

Apigenin 7-glucuronide (purity 99.74%) and luteolin 7-glucuronide (purity 99.98%) were purchased from MedChemExpress (Monmouth Junction, NJ, USA). IPTG, DMSO, D_2_O, TCEP, and other analytical-grade reagents were obtained from Thermo Fisher Scientific (Waltham, MA, USA). CM5 sensor chips, EDC, and NHS were purchased from Cytiva (Marlborough, MA, USA).

### 4.2. Expression and Purification of Recombinant CypD

Recombinant human CypD and uniformly ^15^N-labeled CypD were expressed in *Escherichia coli* BL21 (DE3). The CypD coding sequence was cloned downstream of the ribose-binding protein fusion tag in the pET41 expression vector. Protein expression was induced with IPTG, and the recombinant protein was purified by cation-exchange chromatography followed by size-exclusion chromatography [18]. Protein purity was confirmed to be >95% by SDS-PAGE. Uniformly ^15^N-labeled CypD was prepared using the same protocol, except that the cells were cultured in isotope-enriched minimal medium.

### 4.3. Selection of the Optimal CypD Structure for Molecular Docking

Six experimentally determined CypD crystal structures (PDB IDs: 6R8L, 2Z6W, 4J59, 5CCS, 7R2L, and 3R4G) were retrieved from the Protein Data Bank. Prior to docking, crystallographic water molecules and co-crystallized ligands were removed from each CypD crystal structure using PyMOL (v2.5, Schrödinger LLC, New York, NY, USA). The processed receptor structures were subsequently converted to PDBQT format using the prepare_receptor4.py script from AutoDockTools 1.5.7, which automatically assigned Gasteiger charges and added polar hydrogen atoms. To identify the most suitable receptor structure for virtual screening, receiver operating characteristic (ROC) analysis was performed. Specifically, 80 reported CypD inhibitors were collected from BindingDB (https://www.bindingdb.org/rwd/bind/index.jsp, accessed on 1 June 2023), and approximately 4000 corresponding decoys were generated using the DUD-E server [25]. Three-dimensional structures of all active compounds and decoys were generated and energy-minimized using the MMFF94 force field implemented in RDKit (version 2023.03.1), followed by hydrogen addition. The optimized ligand structures were subsequently converted to PDBQT format using the prepare_ligand4.py script from AutoDockTools 1.5.7. Active compounds and decoys were docked into each CypD structure using AutoDock Vina 1.2 [26]. The receptor was treated as rigid, whereas ligand rotatable bonds were treated as flexible. Docking was performed with an exhaustiveness value of 32, a maximum of 10 binding modes per ligand (num_modes = 10), and an energy range of 3 kcal/mol. The docking grid was centered on the catalytic pocket of CypD, with a box size of 8 × 8 × 8 Å^3^. Compounds were ranked according to their best Vina docking scores. ROC curves were generated from the ranked compounds, and the area under the ROC curve (AUC), hit rate (HR), and enrichment factor (EF) at the top 0.5% of the ranked database were used to evaluate the screening performance of each receptor structure:

(1) $${HR}_{0.5\%}=\frac{{Hit}_{0.5\%}}{Hit}$$

where ${Hit}_{0.5\%}$ is the number of active compounds in the top 0.5% of the database and Hit is the number of all active compounds in the database.

(2) $${EF}_{0.5\%}={HR}_{0.5\%}\times \frac{N}{N_{0.5\%}}$$

where N is the number of all compounds in the database and $N_{0.5\%}$ is the number of all compounds in the top 0.5% of the database.

### 4.4. Virtual Screening by Molecular Docking

Based on the ROC evaluation, the crystal structure with the best screening performance (PDB ID: 6R8L) was selected as the receptor for virtual screening. A total of 484 dietary polyphenols were collected from the Phenol-Explorer database [27]. The ligands were prepared following the same protocol described above, including three-dimensional structure generation, MMFF94 energy minimization, hydrogen addition, and conversion to PDBQT format. Molecular docking was then performed using AutoDock Vina 1.2 under the same docking parameters used for receptor benchmarking. For each compound, the binding mode with the lowest predicted binding energy was retained for ranking and subsequent interaction analysis. The top-ranked compounds were selected for experimental validation.

### 4.5. HSQC NMR Titration

Two-dimensional NMR spectra of CypD were obtained at 25 °C on a Bruker 600 MHz NMR spectrometer (Bruker BioSpin, Billerica, MA, USA) equipped with a cryogenic probe. NMR data were processed and analyzed using Topspin 3.5pl7 and Sparky 3.115. Backbone resonance assignments used for CSP analysis were taken from our previous study. The ^15^N labeled CypD was dissolved in 90/10% H_2_O/D_2_O with 20 mM NaCl, 20 mM Na_2_HPO_4_, 0.3 mM TCEP and 1% DMSO at pH 7.4. Ligands were freshly prepared from DMSO stock solutions and visually inspected to confirm the absence of visible precipitation prior to each titration experiment. One complete series of two-dimensional ^15^N–^1^H HSQC spectra was acquired for each ligand by titrating 100 μM ^15^N-labeled CypD with increasing ligand concentrations from 50 μM to 1 mM while maintaining a constant final DMSO concentration. The chemical shift perturbation (CSP) of CypD for amide ^1^H and ^15^N chemical shifts were calculated using the equation:

(3) $$CSP=\sqrt{100\times ∆H^{2}+∆N^{2}}$$

where ∆H and ∆N are the differences between the chemical shifts in the free and bound forms of CypD, respectively.

Residues with CSP values ranked within the top 10% of all assigned residues were considered significantly perturbed.

### 4.6. SPR Measurements

Surface plasmon resonance (SPR) measurements were performed using a BIAcore 3000 system (GE Healthcare, Chicago, IL, USA) to characterize the binding of apigenin 7-glucuronide and luteolin 7-glucuronide to CypD. CypD was immobilized on a CM5 sensor chip via standard EDC/NHS-mediated amine coupling. Briefly, CypD (0.1 mg/mL, 10 μL) was injected over the activated sensor surface at a flow rate of 5 μL/min, resulting in an immobilization level of approximately 5000 resonance units (RU). A reference flow cell was prepared using the same procedure without CypD immobilization for background correction. Apigenin 7-glucuronide and luteolin 7-glucuronide were diluted in running buffer containing 20 mM Tris–HCl, 150 mM NaCl, and 1 mM TCEP (pH 7.2) to final concentrations of 39, 78, 156, 313, 625, and 1250 μM and injected over the sensor surface at a flow rate of 30 μL/min for 3 min, followed by a 4 min dissociation phase with running buffer. The sensor surface was regenerated with 20 μL of 10 mM glycine–HCl (pH 2.25). All measurements were performed at 25 °C. The resulting sensorgrams were processed using BIAevaluation software (version 4.1.1, GE Healthcare, Chicago, IL, USA) and globally fitted using a two-state binding model to determine the equilibrium dissociation constant (*K_D_*).

### 4.7. Statistical Analysis

Statistical analyses were performed using GraphPad Prism version 9.5 (GraphPad Software, Boston, MA, USA). Nonlinear regression analysis was performed to estimate apparent dissociation constants.

## 5. Conclusions

Overall, this study established and optimized a computational screening and molecular validation workflow for identifying CypD-binding compounds. Through virtual screening of dietary polyphenols, apigenin 7-glucuronide and luteolin 7-glucuronide were identified as candidate CypD ligands. Molecular docking and NMR provided complementary structural and residue-level evidence for their interactions with CypD, while SPR independently confirmed direct binding with *K_D_* values of 66 and 84 μM, respectively. These findings demonstrate the utility of integrating structure-based virtual screening with complementary biophysical approaches for the identification and characterization of CypD-binding compounds and provide a basis for further exploration of natural polyphenol derivatives targeting CypD.

## Figures and Tables

**Table 1:** The AUC, HR_0.5%_ and EF_0.5%_ for the 6 CypD structures.

PDB ID	AUC	HR_0.5%_	EF_0.5%_
6R8L	0.75	0.088	17.8
4J59	0.74	0.013	2.5
2Z6W	0.66	0.063	12.7
5CCS	0.70	0	0
3R4G	0.63	0	0
7R2L	0.63	0	0

**Table 2:** The top five ranked compounds and positive control in docking.

Rank	Compound	Structure	Affinity (kcal/mol)
1	Apigenin 7-glucuronide	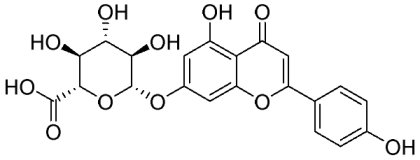	−10.41
2	Positive Control (the 6R8L-bound ligand)	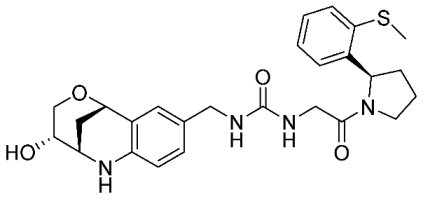	−10.16
3	6″-O-Acetylglycitin	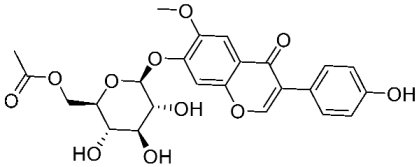	−9.90
4	Luteolin 7-glucuronide	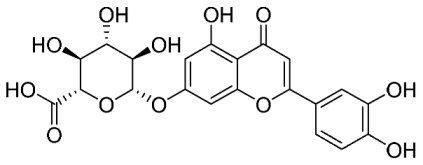	−9.70
5	Ellagic acid glucoside	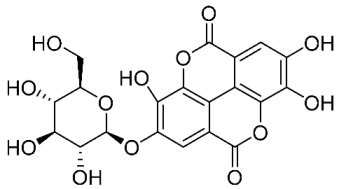	−9.60
6	5,3′,4′-Trihydroxy-3-methoxy-6,7-methylenedioxyflavone 4′-O-glucuronide	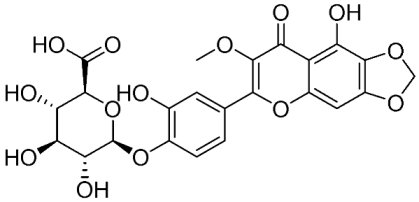	−9.59

## Data Availability

The data supporting the findings of this study are available from the corresponding author upon reasonable request.

## Abbreviations

The following abbreviations are used in this manuscript:

AUC	Area under the receiver operating characteristic curve
CSP	Chemical shift perturbation
CypD	Cyclophilin D
EF	Enrichment factor
HSQC	Heteronuclear single quantum coherence
HR	Hit rate
*K_D_*	Dissociation constant
mPTP	Mitochondrial permeability transition pore
NMR	Nuclear magnetic resonance
PDB	Protein Data Bank
ROC	Receiver operating characteristic
RMSD	Root-mean-square deviation
